# Mutation profile of BBS genes in patients with Bardet–Biedl syndrome: an Italian study

**DOI:** 10.1186/s13052-019-0659-1

**Published:** 2019-06-13

**Authors:** Elena Manara, Stefano Paolacci, Fabiana D’Esposito, Andi Abeshi, Lucia Ziccardi, Benedetto Falsini, Leonardo Colombo, Giancarlo Iarossi, Alba Pilotta, Loredana Boccone, Giulia Guerri, Marica Monica, Balzarini Marta, Paolo Enrico Maltese, Luca Buzzonetti, Luca Rossetti, Matteo Bertelli

**Affiliations:** 1 Magi Euregio, Bolzano, Italy; 2 Imperial College Ophthalmic Research Unit, Western Eye Hospital, Imperial College Healthcare NHS Trust, London, UK; 3 Eye Clinic, Department of Neurosciences, Reproductive Sciences and Dentistry, Federico II University, Naples, Italy; 4 IRCCS – Fondazione Bietti, Rome, Italy; 5 Institute of Ophthalmology, Università Cattolica del Sacro Cuore, Rome, Italy; 6 Fondazione Policlinico Universitario “A. Gemelli”, IRCCS, Rome, Italy; 7 Department of Ophthalmology, San Paolo Hospital, University of Milan, Milan, Italy; 8 Department of Ophthalmology, Bambino Gesù IRCCS Children’s Hospital, Rome, Italy; 9 Special Unit of Auxoendocrinology, Diabetology and Pediatric Genetics, University of Brescia, Spedali Civili di Brescia, Brescia, Italy; 10 Microcitemic Regional Hospital, Brotzu Hospital, Cagliari, Italy; 11 MAGI’S Lab, Rovereto, Italy

**Keywords:** NGS, Bardet-Biedl syndrome, Genetic diagnosis; triallelic inheritance

## Abstract

**Background:**

Bardet–Biedl syndrome (BBS) is a rare inherited multisystemic disorder with autosomal recessive or complex digenic triallelic inheritance. There is currently no treatment for BBS, but some morbidities can be managed. Accurate molecular diagnosis is often crucial for the definition of appropriate patient management and for the development of a potential personalized therapy.

**Methods:**

We developed a next-generation-sequencing (NGS) protocol for the screening of the 18 most frequently mutated genes to define the genotype and clarify the mutation spectrum of a cohort of 20 BBS Italian patients.

**Results:**

We defined the causative variants in 60% of patients; four of those are novel. 33% of patients also harboured variants in additional gene/s, suggesting possible oligogenic inheritance. To explore the function of different genes, we looked for correlations between genotype and phenotype in our cohort. Hypogonadism was more frequently detected in patients with variants in BBSome proteins, while renal abnormalities in patients with variations in BBSome chaperonin genes.

**Conclusions:**

NGS is a powerful tool that can help understanding BBS patients’ phenotype through the identification of mutations that could explain differences in phenotype severity and could provide insights for the development of targeted therapy. Furthermore, our results support the existence of additional BBS loci yet to be identified.

**Electronic supplementary material:**

The online version of this article (10.1186/s13052-019-0659-1) contains supplementary material, which is available to authorized users.

## Background

Bardet-Biedl syndrome (BBS) is a rare inherited, clinically and genetically heterogeneous, multisystemic ciliopathy with various primary and secondary clinical manifestations [1]. Although the common postaxial hexadactyly is evident at birth, in absence of a family history the diagnosis is usually made after the manifestation of ocular involvement. The main features are: retinal dystrophy (cone-rod type) often leading to blindness, upper and lower limbs polydactyly, early-onset truncal obesity, intellectual impairment, hypogonadism and renal abnormalities. A number of secondary features is also described [1]. Life expectancy can be reduced, mainly due to renal failure [2].

**Fig. 1:**
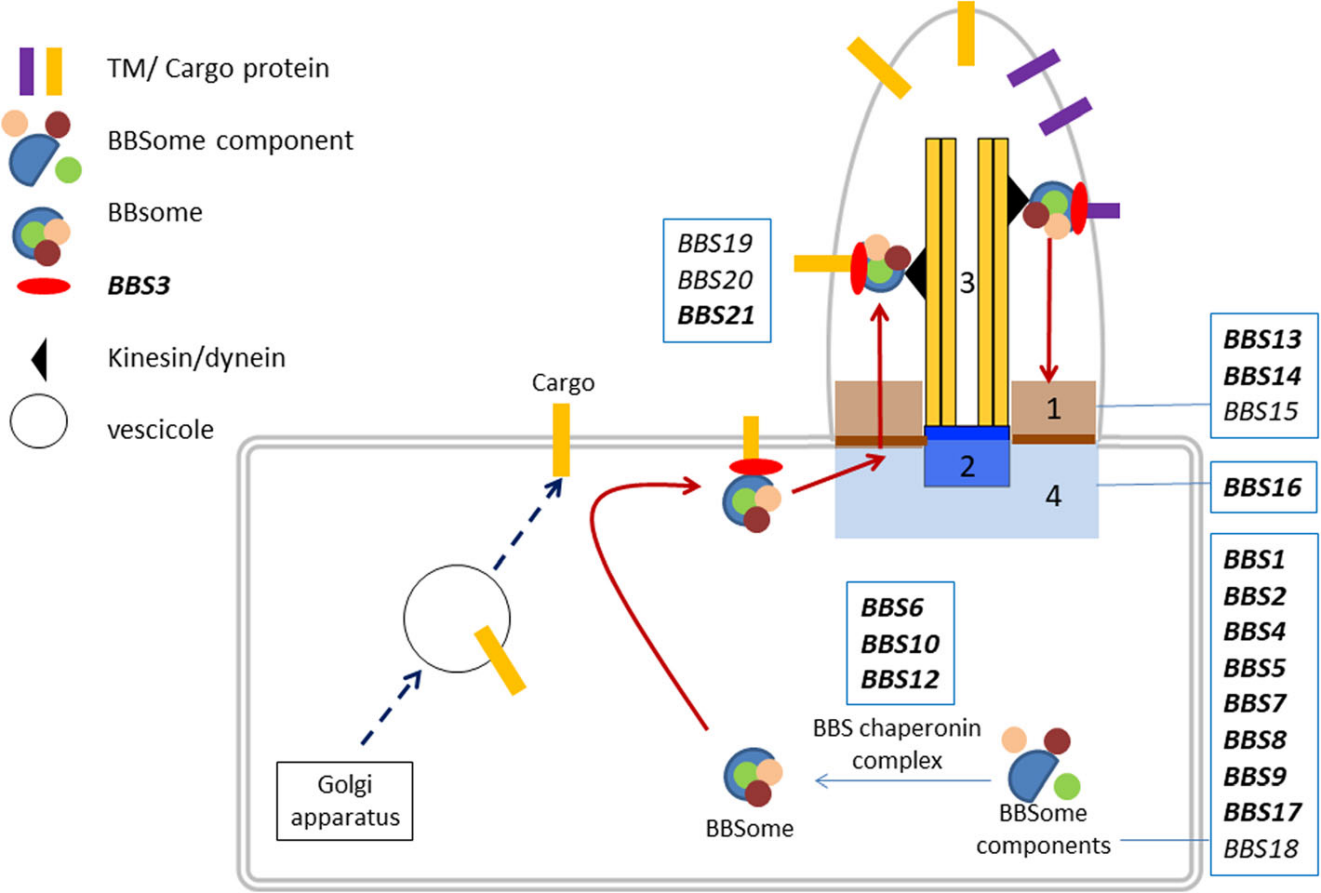
Schematic representation of the cilium and intraflagellar transport. 1. transition zone; 2. basal body; 3. axoneme; 4. pericentriolar area. BBS-chaperonin complex (BBS6, BBS10, BBS12) binds and stabilizes the BBS protein to form the BBSome (BBS1, BBS2, BBS4, BBS5, BBS7, BBS8, BBS9, BBS17, BBS18). BBSome plays a critical role in the regulation of cilia composition and in intraflagellar trafficking. Indeed, transmembrane (TM) and periferal membrane protein are transported in the cilium in a BBSome dependent manner. BBS3 triggers BBSome complex /cargo proteins interaction and their transition across the control barrier (transition zone - BBS13, BBS14, BBS15) into the cilium. In bold, genes included in our NGS panel

Twenty-one different loci (BBS1-BBS21) have been associated with this syndrome. The genes mostly code for proteins forming the core BBSome complex (BBS1, BBS2, BBS4, BBS5, BBS7, BBS8 and BBS9) or part of a BBS chaperone complex (BBS6, BBS10 and BBS12) which plays an essential role in the stabilization and regulation of the BBSome [3, 4]. Other genes code for proteins with roles in the localization and activation of BBSome (ARL6) or BBSome entry into cilia (BBS17) or are associated with the BBSome complex (BBS14) (Fig. 1 and Additional file 1: Table S1). The functions of some of the proteins are not fully understood. BBSome is a stable protein complex that functions in the biogenesis and maintenance of the primary cilium (Fig. 1), a structure that is ubiquitously expressed and highly conserved through evolution, and in modulating ciliary protein trafficking. Primary cilia serve as sensors of the extracellular environment, they receive and transduce signals from light, chemical, or mechanical stimuli, [5] in addition they play a role in several signalling pathways important for development and tissue homeostasis, their alteration results in abnormalities and multiorgan disfunctions. Many different pathways have been linked to the primary cilium, including Hedgehog, Wnt, Notch, Hippo, GPCR, PDGF, mTOR, and TGF-beta [6].

For many years BBS was considered an autosomal recessive disease, but recently evidence suggesting complex digenic triallelic inheritance has been described [7–9]. This could partly explain the large, phenotypic heterogeneity found in BBS patients, both inter- and intrafamilial [10].

There is currently no treatment for BBS, but some of the co-morbidities can be managed. Precise identification of the causative gene(s) is therefore a fundamental step toward a personalized therapeutic approach and management of genotype-related conditions [8, 11–17]. Thus, genetic analysis and accurate phenotyping are fundamental for stratifying patients and addressing appropriate therapy. In our study we analysed the mutation spectrum in a cohort of 20 Italian patients with BBS, investigating the tri-allelic hypothesis and analysing genotype-phenotype correlation.

## Materials and methods

### Patients

Twenty caucasian patients diagnosed in different hospital across Italy with Bardet-Biedl syndrome were retrospectively included in the study. The mean (±SD) age was 29 ± 17.1 (range 9–63) years and the male/female ratio was 13:7. The mean age at diagnosis was 5.4 ± 7.2 (range 0–24) years. No consanguinity in their families was reported [except for two probands that stated a distant kinship]. Clinical diagnosis of BBS was made according to the accepted criteria [1]. Genetic testing was performed on germline DNA extracted from either saliva or blood of the proband.

### Mutation analysis

A custom-made oligonucleotide probe library was designed to capture all coding exons and flanking exon/intron boundaries (±15 bp) of 18 genes known to be associated with Bardet-Biedl syndrome (Additional file 1: Table S1 and Additional file 2: for protocol details). DNA from the proband was analysed. Identified variants with likely clinical significance (pathogenic, likely pathogenic and of unknown significance according to the ACMG guideline) [18] were confirmed by bidirectional Sanger sequencing on a CEQ8800 Sequencer (Beckman Coulter). Segregation in family members was performed for variants identified in the proband in heterozygous state in order to confirm that the variants were in trans (yes in column Seg in Table 1).

**Table 1:** Bardet-Biedl syndrome patients with resolved genotype

	Sex	Seg	Gene	Ex/ int	Nucleotide substitution	Protein substitution	Het/Homo	Type	Score [18]	Ref	RS	MAF
1	M	Yes	BBS2	ex9	c.1015C > T	p.(Arg339*)	Het	nonsense	P	[14]	rs193922710	N/A
			BBS2	ex9	**c.1062C > G**	p.(Asn354Lys)	Het	missense	P			
2	M	Yes	BBS10	ex2	c.1091del	p.(Asn364Thrfs*5)	Het	frameshift	P	[15]	rs727503818	0.00005
			BBS10	ex2	c.1677del	p.(Tyr559*)	Het	nonsense	P	[4]		
3	M		BBS7	ex8	**c.763A > T**	p.(Lys255*)	Homo	nonsense	P			
4	F		BBS2	ex8	c.814C > T	p.(Arg272*)	Homo	nonsense	P	[16, 17]		
			BBS12	ex2	c.116 T > C	p.Ile39Thr	Het	missense	fSNP	[19]	rs138036823	
			INPP5E	ex1	c.532G > A	p.Val178Met	Het	missense	VUS			
5	M		BBS10	ex2	c.271dup	p.(Cys91Leufs*5)	Homo	nonsense	P	[4, 20, 21]	rs549625604	0.0007
6	F		BBS12	ex2	c.1063C > T	p.(Arg355*)	Homo	nonsense	P	[22]	rs121918327	0.00002
			BBS1	ex12	**c.1016A > T**	p.(His339Leu)	Het	missense	VUS			
7	F	Yes	BBS10	ex2	c.641 T > A	p.(Val214Glu)	Homo	missense	P	[23]		
8	M	Yes	BBS10	ex2	c.1676dup	p.(Tyr559*)	Het	nonsense	P	[24]		
			BBS10	ex2	c.962A > G	p.(Tyr321Cys)	Het	missense	LP	[23]		
9	F		BBS12	ex2	c.1531_1539del	p.(Gln511_Gln513del)	Homo	inframe del	P	[4, 19]	rs752762669	
10	M	Yes	BBS1	ex1	c.46A > T	p.(Ser16Cys)	Het	missense	LP		rs772917364	0.008458
			BBS1	ex13	**c.1285dup**	p.(Arg429Profs*72)	Het	frameshift	P			
			BBS10	ex2	c.765G > A	p.(Met255Ile)	Het	missense	LB	[25]	rs139658279	
			BBS14	ex10	c.829G > C	p.(Glu277Gln)	Het	missense	VUS	[26]	rs45502896	
11	M	Yes	BBS4	int5	c.332 + 2_332 + 3insTT		Het	Insertion	P	[27]	rs753360929	
			BBS4	ex13	c.1091C > A	p.(Ala364Glu)	Het	missense	P	[28]	rs28938468	
			BBS8	ex4	c.254A > G	p.(Lys85Arg)	Het	missense	VUS		rs150880478	
			BBS2	ex9	c.986 T > C	p.(Met329Thr)	Het	missense	VUS		rs201146063	
12	F	Yes	BBS6	ex5	**c.1235G > T**	p.(Cys412Phe)	Homo	missense	LP			

Never previously reported nucleotide substitutions are in bold

*Abbreviations*: *M* male, *F* female, *seg* segregation performed, *ex* exon, *int* intron, *dup* duplication, *del* deletion, *ins* insertion, *het* heterozygous, *homo* homozygous, *P* pathogenic, *LP* likely pathogenic, *LB* likely benign, *VUS* variant unknown significance, *fSNP* functional single nucleotide polymorphism, *Ref* references, *RS* dbSNP accession number, *MAF* minor allele frequency

### Statistical analysis

The relation between two class group variables was assessed by Fisher exact test.

## Results

The 20 patients with Bardet-Biedl syndrome enrolled in this study were screened using a panel of 18 genes associated with the disease. We obtained the following results. No variants could be found in eight patients whose characteristics are described in Additional file 1: Table S3). We defined the causal variants in 12 patients (60%) (Table 1); 5 patients were confirmed to have a compound heterozygous variant in a *BBS* gene, while 7 patients where homozygous for the causative variant. In four cases the pathogenic variants were novel. We identified a novel compound heterozygous variant in *BBS1* c.1285dup (p.(Arg429Profs*72); a likely pathogenic novel variant affecting the conserved residue 354 in the functional domain of *BBS2* (c.1062C > G; p.(Asn354Lys)); a pathogenic new homozygous nucleotide change in *BBS7* that leads to a stop codon in position 255, c.763A > T, and a likely pathogenic homozygous substitution c.1235G > T in *BBS6*, leading to the change p.(Cys412Phe). The novel variants were scored as pathogenic or likely pathogenic according to Richards et al. classification [18]. Four of the 12 patients in which the causative gene had been identified, also had potentially pathogenic variants in additional BBS genes suggesting oligogenic inheritance and a possible modifier effect. Three out of 4 patients presented variants in two additional genes, while patient #6 presented a variant with unknown significance (VUS) in *BBS1* in addition to the “principal variants” in *BBS12.* Patient #4 presented “principal mutations” in *BBS2* and additional functional polymorphism in *BBS12* and a variant with unknown significance in *INPP5*; patient #10 presented additionally to the principal variant in *BBS1*, variants in *BBS10* and *BBS14*, the first likely benign and the second with unknown significance; patients #11 with compound heterozygous variants in *BBS4*, presented additionally VUS in *BBS8* and *BBS2*. Unlikely, we cannot document any influence on the phenotype severity in patient carrying additional variants in heterozygous state due to the small cohort of patients analysed and to the fact that all the variants were missense and with an uncertain role in disease manifestation.

In our cohort, we had a prevalence of patients harbouring genetic variants in *BBS10* (*N* = 4), two patients with variants in *BBS2* and two in *BBS12*. The remaining four patients had their causative pathogenic variants in each of the following genes: *BBS1*, *BBS4*, *BBS6*, *BBS7*.

The clinical characteristics of positive patients are reported in Table 2. Patients were predominantly males, and all were clinically diagnosed during childhood or adolescence (median age 21 months). All patients with a molecular diagnosis had polydactyly of one or both hands or feet, in most cases hexadactyly of feet as well as hands (*N* = 6) (Table 2 and Additional file 1: Table S2). All patients had cone-rod dystrophy / retinitis pigmentosa (CRD/RP), except one who had myopia and cataract (Additional file 1: Table S2), highlighting the fact that clinical diagnosis of BBS is often made when patients manifest ocular involvement. Other frequent clinical characteristics in our cohort were intellectual disability, hypogonadism, obesity and renal abnormalities (Additional file 1: Table S2). The cohort of negative patients was not statistically significantly different from the cohort of molecularly resolved patients (Additional file 1: Table S2).

**Table 2:** Clinical manifestations of Bardet-Biedl syndrome patients with resolved genotype

ID	Main gene	Sex	Onset	Ocular disease	BMI (Kg/m2)	Intellectual disabilities affecting:			Hexadactyly		Additional features					Consanguineity	Mean depth coverage (X)	Target coverage at 25X
						Cognitive skills	Language skills	Motor skills	Hands	Feet	Renal anomalies	Hepatic steatosis	Hypercholesterolemia	Hypogonadism	Other			
1	BBS2	M	13 yrs	RP	O	no	no	no	yes, right	yes, both	no	no	no	yes	yes		197.37	97.3
2	BBS10	M	6 yrs	RP	O	no	no	no	yes, both	no	yes	N/K	yes	N/K		no	190.6	96.5
3	BBS7	M	6mo	CRD	O	mild	mild	mild	yes, both	yes, both	yes	N/K	N/K	yes		N/K	214.8	97.2
4	BBS2^a^	F	2 yrs	CRD + HM	37, O	mild	mild	mild	yes, both	yes, both	no	yes	no	yes	yes	no	264.13	97.5
5	BBS10	M	since birth	HM + RP	24.9, OW	yes	yes	yes	yes, both	yes, both	yes	no	no	no			184.6	96.8
6	BBS12^a^	F	since birth	RP	no	yes	yes	yes	no	yes, both	yes	no	no	yes		no	226.11	97.2
7	BBS10	F	18mo	RP	31.6, O	no	no	no	yes, left	no	yes	no	no	no			283.18	98.3
8	BBS10	M	17 yrs	RP	30.2, O	yes	no	no	no	yes, both	no	no	no	no		no	181.03	97.0
9	BBS12	F	5 yrs	HM + C	29.3, OW	no	no	no	yes, both	yes, both	no	no	no	no			150.6	99.0
10	BBS1^a^	M	28mo	RP	OW	no	no	no	no	yes, both	no	no	no	yes		no	110.2	91.2
11	BBS4^a^	M	6mo	RP	26.4, OW	yes	yes	yes	yes, both	yes, both	no	yes	no	yes		no	160.7	92.5
12	BBS6	M	18mo	RP	30, O	no	yes	no	yes, right	yes, both	no	no	no	yes		distant kinship	197.61	93.2
Sister of 12	BBS6	F	18mo	RP	28.0, OW	no	no	no	no	yes, left	no	no	no	no		distant kinship	–	–

*Abbreviations*: *M* male, *F* female, *yrs*. years, *mo* months, *RP* retinitis pigmentosa, *CRD* cone-rod dystrophy, *HM* high myopia, *C* cataract, *O* obese, *OW* overweight, *N/K* not known

^a^indicates an additional mutant BBS gene

## Discussion

Bardet-Biedl syndrome, a ciliopathy with autosomal recessive/oligogenic inheritance shows high clinical variability and genetic heterogeneity. Although the postaxial polydactyly is evident at birth, clinical diagnosis is usually made when patients complain of visual distress. Genetic testing can help to confirm the diagnosis and enable prompt and effective clinical management. The molecular characterization of patients in addition can help to predict a grade of severity and to identify carrier’s family members with potential benefits of counselling. The targeted sequencing also has the advantage of allowing the identification of modifiers or genes with an epistatic effect that can shed light on phenotypic variability between and within families. Furthermore the molecular diagnosis can be fundamental for personalized medicine, aiming at the identification of patients potentially suitable for enrolment in any appropriate clinical trial that may become available in the future.

In this direction, recent advances in gene therapy and personalized medicine have enabled significant advances in the development of potential therapies for BBS patients, although the number of genes involved and the variety of mutations identified in patients, often in the same family, has increased the challenge faced by researchers [29].

Our study was performed to increase the BBS mutation spectrum in a cohort of 20 unrelated Italian patients with BBS. Genetic diagnosis was obtained in 12/20 patients using a NGS targeted approach analysing simultaneously the 18 most frequently mutated genes associated with BBS to increase the detection rate and the understanding of the BBS phenotype through discovery of additional mutations in BBS genes that could explain differences in phenotype severity.

The study revealed *BBS10* gene mutations in a majority of our cohort (33%), in accordance with the percentages already reported in the literature [4]. Interestingly, we found just one patient with variants in *BBS1*, the most frequently detected gene in BBS patients [4, 30–32].

We identified a novel variant in *BBS1* patient #10 c.1285dup (p.(Arg429Profs*72)) defined as pathogenic that segregates with phenotype together with c.46A > T (p.(Ser16Cys), defined as likely pathogenic.

A new pathogenic variant in *BBS2* affecting a conserved residue in the functional domain of BBsome protein (c.1062C > G; p.(Asn354Lys)) was found in compound heterozygous state in patient #1 together with the known pathogenic variant p.(Arg339*). A new homozygous nucleotide change in *BBS7* that leads to a stop codon in position 255, c.763A > T, was identified in patient #3. *BBS1*, *BBS2* and *BBS7* share a partially overlapping portion of a functional domain, mutation of which results in the same disease phenotype [30]. New pathogenic variants of *BBS2* and *BBS7* lie in this portion. The variant in *BBS7* is noteworthy, since very few Bardet-Biedl cases are reported in the literature. Indeed, only 35 variants [33] in this gene are listed in the Human Gene Mutation Database (HGMD, https://portal.biobase-international.com/cgi-bin/portal/login.cgi). A homozygous substitution c.1235G > T in *BBS6*, leading to p.(Cys412Phe), was also identified in an affected sibling of proband #12. Interestingly, the clinical severity of the phenotypes of the two siblings was different (Table 2), suggesting that these variants show intrafamilial variable expressivity or that the patient’s genetic background strongly influences phenotype.

Interestingly, four of the twelve patients in which the causative gene had been identified, also had additional variants in BBS genes suggesting oligogenic inheritance and a possible modifier effect. However, we cannot document any influence on the phenotype severity in patient carrying additional variants in heterozygous state due to the small cohort of patients analysed and to the fact that all the variants were missense and with an uncertain role in disease manifestation. Our results are concordant with the results obtained also by other authors: the impact of the additional heterozygous variant remains elusive since it is very difficult to determine the contribution of the third allele to the phenotype. Available studies compare different kind of mutations, with different impact on the protein in small cohorts with high genetic heterogeneity [32, 34]. Moreover, in our cohort, we do not present family members with difference in the genetic background that could help shed light on the triallelic inheritance hypothesis [30].

In order to explore the function of different genes involved in BBS we looked for possible correlations in our cohort dividing patients with a molecular diagnosis with variants in BBSome genes or with variants in BBSome chaperonin genes. The clinical phenotype spectrum is wide (different type of mutations on different genes) and we did not observe any correlation between characteristic of patients probably due to the small number of patients analysed. This limits the possibility to highlight genotype-phenotype correlation with a statistical significance. Previous study have not identified any correlation between individual genotypes and phenotype [35, 36]. Interestingly, Billingsley et al. stated that patients with mutations in *BBS10* or *12* had a similar phenotype, supporting our sorting of patients [33].

Characteristics such as obesity and intellectual impairment or retinal degeneration affected patients with mutations in genes of the BBSome or coding for BBSome chaperonin with the same frequencies. Hypogonadism (manifesting as genital anomalies in females and small penis buried in adipose tissue with undescended testes in males [11]) was more frequent in patients with variants in BBSome protein, whereas renal abnormalities were mostly present in patients with variations in BBSome chaperonin genes [12] (Table 3). Patients with renal anomalies should therefore be screened mainly in *BBS10*, *BBS12, BBS6* and those with hypogonadism for variants in BBSome genes. Clinicians should also closely monitor patients harbouring mutations in *BBS10, BBS12, BBS6* to favour early detection of those with renal anomalies, at risk of kidney failure and sudden death.

**Table 3:** Genotype/Phenotype correlations

	BBSome	BBSome chaperonin	Fisher’s exact test
Obesity	3/5 (60%)	4/7 (57.1%)	*P* = 1.0
Intellectual impairment	3/5 (60%)	4/7 (57.1%)	P = 1.0
Renal abnormalities	1/5(20%)	4/7 (57.1%)	*P* = 0.6
Hypogonadism	5/5 (100%)	2/7 (28.5%)	*P* = 0.35

In our cohort, hexadactyly was found in all patients with a molecular diagnosis. Confirming the results of Beales et al., polydactyly of the toes was more common than that of the fingers in our cohort (11vs9), and both feet more often had polydactyly than both hands (90% vs 66%) [13].

## Conclusion

In conclusion, our results demonstrate that NGS panels are a fast and effective way of obtaining high diagnostic yields in diseases, such as BBS, caused by mutations in many genes. They also provide information on other mutant genes in addition to the causative one. Genetic testing can provide insights into the pathways involved in the disease and aid the development of targeted therapy, which needs to begin with the first eye symptoms, before extensive photoreceptor damage. When feasible, NGS should be considered the elective method of genetic testing to confirm any hypothesis of tri-allelic inheritance. Our results are further evidence that BBS is extremely heterogeneous: by describing four new variants we extend the mutational spectrum of known BBS genes and contribute information on genotype-phenotype correlations. Finally, the fact that 40% of our patients did not have any mutation in the 18 known screened genes, leads us to the consideration that although being the best available approach, targeted NGS do not allow the detection of (i) deeply intronic variants that could have an effect either on the splicing or expression of the genes; (ii) large gene rearrangements nor (iv) the analysis of additional responsible loci yet to be identified. In addition given the fact that there is a significant overlap between clinical sign and mutated genes within BBS and other ciliopathies such as McKusick-Kaufman syndrome (MKKS; OMIM 604896), Alstrom syndrome (ALMS; OMIM 203800), Meckel-Gruber syndrome (MKS; OMIM 249000, 603294, 607361, 611134), and Joubert syndrome (JBTS; OMIM 213300, 608,091, 608629, 609583, 610688, 611560, 612291, 612285, 300804) we cannot exclude that our negative patients presented variants in genes more associated to the other syndromes and that were not sequenced.

## Additional files

Additional file 1: **Table S1.** List of genes in the NGS panel. **Table S2.** Distribution of BBS diagnostic criteria in patients with molecular diagnosis. **Table S3.** Clinical manifestations of Bardet-Biedl syndrome patients with unresolved genotype. Abbreviations: M, male; F, female; yrs., years; mo, months; RP retinitis pigmentosa; CRD, cone-rod dystrophy; HM, high myopia; O, obese; OW, overweight; N/K, not known. (DOCX 28 kb)

Additional file 2: Mutation analysis. The DNA probe set was designed using specific Illumina DesignStudio online tool (https://designstudio.illumina.com/). (DOCX 14 kb)

## Data Availability

The novel variant identified during the current study are available in the Clinvar repository (SUB4899621).

## Abbreviations

BBS: Bardet-Biedl syndrome
CRD: Cone-rod dystrophy.
del: Deletion
dup: Duplication
ex: Exon
F: Female
fSNP: Functional single nucleotide polymorphism
het: Heterozygous
homo: Homozygous
ins: Insertion
int: Intron
LB: Likely benign
LP: Likely pathogenic
M: Male
MAF: Minor allele frequency
NGS: Next generation sequencing
P: Pathogenic
Ref: References
RP: Retinitis pigmentosa
RS: dbSNP accession number
seg: Segregation performed
VUS: Variant unknown significance
