# Supplementary material for: Mutation profile of BBS genes in patients with Bardet–Biedl syndrome: an Italian study
Source: Ital J Pediatr. 2019 Jun 13;45:72. doi: 10.1186/s13052-019-0659-1 (PMC6567512; doi:10.1186/s13052-019-0659-1)
Supplement: Supplementary file 1 — Table S1. List of genes in the NGS panel. Table S2. Distribution of BBS diagnostic criteria in patients with molecular diagnosis. Table S3. Clinical manifestations of Bardet-Biedl syndrome patients with unresolved genotype. Abbreviations: M, male; F, female; yrs., years; mo, months; RP retinitis pigmentosa; CRD, cone-rod dystrophy; HM, high myopia; O, obese; OW, overweight; N/K, not known. (DOCX 28 kb) [file 13052_2019_659_MOESM1_ESM.docx]

| HGNC | BBS Gene | chromosome; ref seq; OMIM gene | Classification | Role | OMIM BBS |
| --- | --- | --- | --- | --- | --- |
| *BBS1* | *BBS1* | chr11; NM_024649; 209901 | BBSome | Member of the BBSome | 12118255 |
| *BBS2* | *BBS2* | chr16; NM_031885; 606151 | BBSome | Member of the BBSome | 11285252 |
| *ARL6* | *BBS3* | chr3; NM_177976; 608845 | BBSome Associated | Small GTPase, BBSome assembly and traffic | 15314642 |
| *BBS4* | *BBS4* | chr15; NM_033028; 600374 | BBSome | Member of the BBSome | 11381270 |
| *BBS5* | *BBS5* | chr2; NM_152384; 603650 | BBSome | Member of the BBSome | 15137946 |
| *MKKS* | *BBS6* | chr20; NM_018848; 604896 | BBSome chaperonin | Assist in BBSome assembly | 10973238 |
| *BBS7* | *BBS7* | chr4; NM_176824; 607590 | BBSome | Member of the BBSome | 12567324 |
| *TTC8* | *BBS8* | chr14; NM_198309; 608132 | BBSome | Member of the BBSome | 14520415 |
| *PTHB1* | *BBS9* | chr7; NM_198428\|NM_001033605; 607968 | BBSome | Member of the BBSome | 16380913 |
| *BBS10* | *BBS10* | chr12; NM_024685; 610148 | BBSome chaperonin | Assist in BBSome assembly | 20805367 |
| *TRIM32* | *BBS11* | chr9; NM_012210; 602290 | Non-BBSome | Processor of BBS2 | 16606853 |
| *BBS12* | *BBS12* | chr4; NM_152618; 610683 | BBSome chaperonin | Assist in BBSome assembly | 17160889 |
| *MKS1* | *BBS13* | chr17; NM_017777; 609883 | Non-BBSome | Transition zone organization | 18327255 |
| *CEP290* | *BBS14* | chr12; NM_025114; 610142 | BBSome Associated | Transition zone organization. Assembly and ciliary entry of the BBSome | 18327255 |
| *SDCCAG8* | *BBS16* | chr1; NM_006642; 613524 | Non-BBSome | Regulates accumulation of pericentriolar material | 20835237 |
| *LZTFL1* | *BBS17* | chr3; NM_020347; 606568 | BBSome Associated | BBSome interacting protein | 22510444 |
| *C8orf37* | *BBS21* | chr8; NM_177965; 614477 | Non-BBSome | Protein trafficking | 27008867 |
| *INPP5E* |  | chr9; NM_019892; 613037 | Other | Regulates the phosphoinositide composition of the cilium membrane | - |

Additional file 1: Table S1. List of genes in the NGS panel

| Criteria | Patients with mutations  # (%) | Patients without mutations  # (%) | Fisher's exact test |
| --- | --- | --- | --- |
| Polydactyly | 12/12 (100%) | 2/6 (33.3%) | **P = 0.0007** |
| CDR/RP | 11/12 (94.6%) | 7/8 (87.5%) | **P = 1.0000** |
| Intellectual impairment | 7/12 (66%) | 3/6 (50%) | **P = 1.0000** |
| Obesity | 7/12 (66%) | 3/6 (50%) | **P = 1.0000** |
| Hypogonadism | 7/11 (63.6%) | 2/7 (28.6%) | **P = 0.3348** |
| Renal abnormalities | 5/12 (41.6%) | 4/6 (66.7%) | **P = 0.6199** |

Additional file 1: Table S2. Distribution of BBS diagnostic criteria in patients with or without molecular diagnosis

| ID | Sex | Age at diagnosis | Ocular disease | BMI (Kg/m^2^) | Intellectual disabilities affecting: | | | Hexadactyly | | Additional features | | | | | Consanguinity | Mean depth coverage (X) | Target coverage at 25X |
| --- | --- | --- | --- | --- | --- | --- | --- | --- | --- | --- | --- | --- | --- | --- | --- | --- | --- |
|  |  |  |  |  | **Cognitive skills** | **Language skills** | **Motor skills** | **Hands** | **Feet** | **Renal anomalies** | **Hepatic steatosis** | **Hypercholesterolemia** | **Hypogonadism** | **Other** |  |  |  |
| 13 | M | N/K | RP | N/K | N/K | N/K | N/K | N/K | N/K | N/K | N/K | N/K | N/K | N/K | N/K | 202.34 X | 97.83% |
| 14 | M | 18yrs | RP | N/K | yes | yes | no | yes, both | yes, both | yes | no | no | no | no | no | 155.28 X | 96.68% |
| 15 | M | since birth | RP | O | yes | no | no | no | no | yes | yes | no | no | yes | no | 267.71 X | 97.58% |
| 16 | M | infancy | RP | O | no | no | no | no | no | yes | yes | yes | yes | no | no | 143 X | 98.10% |
| 17 | F | 2yrs | HM | O | N/K | N/K | N/K | no | yes, both | no | no | no | no | yes | distant kinship | 95.10 X | 91.95% |
| 18 | F | 6mo | CRD | 30.5 | yes | yes | yes | no | no | no | no | yes | no | N/K | no | 281.95 X | 93.58% |
| 19 | F | 24yrs | RP | 30.1 | no | no | yes | no | no | yes | yes | yes | no | yes | no | 126.6 X | 96.7% |
| 20 | M | 6yrs | RP | OW | N/K | N/K | N/K | N/K | N/K | N/K | N/K | N/K | yes | N/K | N/K | 136.8 X | 96.8% |

Additional file 1: Table S3 Clinical manifestations of Bardet-Biedl syndrome patients with unresolved genotype. Abbreviations: M, male; F, female; yrs, years; mo, months; RP retinitis pigmentosa; CRD, cone-rod dystrophy; HM, high myopia; O, obese; OW, overweight; N/K, not known.
