# Supplementary material for: Mutation profile of BBS genes in patients with Bardet–Biedl syndrome: an Italian study
Source: Ital J Pediatr. 2019 Jun 13;45:72. doi: 10.1186/s13052-019-0659-1 (PMC6567512; doi:10.1186/s13052-019-0659-1)
Supplement: Supplementary file 2 — Mutation analysis. The DNA probe set was designed using specific Illumina DesignStudio online tool (https://designstudio.illumina.com/). (DOCX 14 kb) [file 13052_2019_659_MOESM2_ESM.docx]

Additional file 2: Matherials and Methods

Mutation analysis

The DNA probe set was designed using specific Illumina DesignStudio online tool (<http://designstudio.illumina.com/Home/SelectAssay/>). Briefly, in-solution Nextera Rapid Capture target enrichment was performed according to the manufacturer’s protocol. 50 ng of genomic DNA was simultaneously fragmented and tagged by Nextera transposon-based shearing technology. Limited cycle PCR was carried out to incorporate specific index adaptors in each sample library. 500 ng of each indexed DNA library was combined with the 12-plex library pool and then hybridized with target-specific biotinylated probes. The libraries were subsequently captured using streptavidin magnetic beads and underwent a second round of hybridization, capture, PCR amplification and PCR clean-up. The final enriched pooled libraries, with sizes mainly distributed between 500 and 600 bp, were quantified using Qubit (Invitrogen, Carlsbad, CA, USA) and sample quality was verified using an Agilent 2100 BioAnalyzer (Agilent Technologies, Palo Alto, CA). 150 bp paired-end read sequencing was performed on a MiSeq personal sequencer (Illumina, San Diego, CA) according to the manufacturer's instructions. Twenty-four libraries (one for every sample) were loaded on the MiSeq with a V3 kit, obtaining an average yield of 10 Gb and 32 M total reads per run. Raw data in fastq format was analysed using an in-house diagnostic pipeline to generate the final set of sequence variants.
